# Generating a stratocumulus-like cloud top in a convection-cloud chamber

**DOI:** 10.1073/pnas.2519791123

**Published:** 2026-03-12

**Authors:** Aaron Wang, Fan Yang, Mikhail Ovchinnikov, Steven K. Krueger, Raymond A. Shaw

**Affiliations:** ^a^Atmospheric, Climate, and Earth Sciences Division, Pacific Northwest National Laboratory, Richland, WA 99354; ^b^Environmental Science and Technologies Department, Brookhaven National Laboratory, Upton, NY 11973; ^c^Department of Atmospheric Sciences, University of Utah, Salt Lake City, UT 84112; ^d^Department of Physics, Michigan Technological University, Houghton, MI 49931

**Keywords:** entrainment, stratocumulus cloud, cloud chamber, numerical simulation

## Abstract

Stratocumulus-topped boundary layers play a crucial role in influencing daily weather and earth energy balance. Entrainment at the stratocumulus cloud top affects the cloud’s lifetime, precipitation, and radiative properties, but our understanding remains limited due to the lack of resolution in both field observations and numerical simulations. A recently proposed convection-cloud chamber with detailed control of sidewall temperatures can provide a unique opportunity to explore this mechanism in a laboratory setting. In this work, we use numerical simulations to demonstrate that this design can produce a cloud top that mimics the entrainment interfacial layer in a stratocumulus cloud. Our results show that a steady-state cloud can be formed by cooling the lower portions of the sidewalls and warming the bottom surface, while a temperature inversion at the cloud top can be generated by keeping the upper sidewalls and top surface warmer than the bottom. The turbulent kinetic energy profile and budget are similar to those found in a convective boundary layer, and inhomogeneous mixing near the cloud top can be observed. These findings significantly enhance the scientific value of constructing the tall convection-cloud chamber.

## Significance

Entrainment plays a critical role in determining the lifetime and precipitation of stratocumulus clouds, which, in turn, influence regional weather patterns and earth energy balance. Despite its importance, cloud-top entrainment remains a significant source of uncertainty, particularly in coarse-resolution atmospheric models. To date, no laboratory facility has been developed to experimentally study cloud-top entrainment, but a future convection-cloud chamber could provide this unique capability. Using numerical simulations, we demonstrate how a stratocumulus-like cloud top can be replicated through the precise control of wall conditions in a cloud chamber. Additionally, we explore potential experiments and their expected outcomes. Our findings strongly support the development of such a facility and highlight its potential benefits.

Stratocumulus clouds cover a significant portion of the Earth’s surface, influencing regional weather and global climate. Entrainment is the mixing process at the top of the stratocumulus-topped boundary layer (STBL), and it plays a critical role in determining cloud lifetime, precipitation, and radiative properties. How entrainment is represented can influence whether stratocumulus clouds break up, potentially leading to an extreme climate response to increased carbon dioxide (1). Despite its significance, our understanding of entrainment remains limited (2). One of the main reasons is the difficulty in observing fine-scale structural details near the cloud top, which can be as thin as a few meters (e.g., refs. 3 and 4). Field campaigns and numerical simulations of the STBL often lack the resolution needed to capture these details. Specifically, aircraft measurements typically involve horizontal averaging over large spatial scales and fail to resolve local turbulent and microphysical structures, even during vertical profiling. Much higher-resolution vertical profiles have been acquired using helicopter-borne instruments, revealing strong local variability in microphysical properties and entrainment rates (5). However, even these aircraft-based measurements provide only snapshots, limiting our ability to study the entrainment process in detail. Regarding numerical simulations, large-eddy simulations (LES) can resolve the dominant turbulent eddies, but they still struggle to quantitatively predict cloud properties due to challenges in resolving the sharp temperature inversion and detailed entrainment processes (6).

Compared to field measurements and simulations of the entire STBL, laboratory experiments with various fluid layers provide well-defined conditions for exploration and sensitivity tests. Willis and Deardorff and Deardorff and Willis used thermally stratified water in a tank with bottom plate heating to study the growth of the boundary layer (7, 8). Their experiment provides one of the first controlled, quantitative demonstrations of how entrainment and turbulent mixing shape the structure and growth of the convective boundary layer. Sayler and Breidenthal conducted experiments on interfacial convection by radiatively heating the bottom of an upper opaque layer to induce entrainment (similar to inverted cloud-top cooling in the real STBL) (9). Their experiments revealed inverted cusps and hummocks at the interface, resembling structures observed at stratocumulus cloud tops. They demonstrated that the speed of cusps propagating along the interface is proportional to the convective velocity, showing how large eddies influence small-scale structures and ultimately drive entrainment. However, how these entrainment details affect the microphysics of a stratocumulus cloud remains unknown, as no laboratory facility capable of simultaneously incorporating both the layer interface and the cloud droplets has been developed.

Recent advances in cloud chamber research have significantly improved our understanding of aerosol–cloud interactions under turbulent conditions. A notable example is the Pi Convection-Cloud Chamber (10), which has been used to explore various phenomena, including cloud–aerosol–turbulence interactions (11), aerosol-mediated glaciation in mixed-phase clouds (12, 13), and the cloud microphysical response to entrainment (14, 15). Regarding entrainment, the Pi Chamber has provided insights into locally inhomogeneous mixing that can appear as homogeneous mixing when results for various entrainment rates are composited together (14). Additionally, the entrainment experiment reveals the significant role of gravitational sedimentation when aerosols are present in entrained air (16). Numerical simulations of localized entrainment zones within a cloud chamber have proven valuable for interpreting these experimental results (15). Nevertheless, in existing experiments, entrainment has been imposed by forcing air into the cloud, rather than being driven by turbulence across the interface between the STBL and the free atmosphere.

Building on the success of the Pi Chamber, a tall convection-cloud chamber with flexible control of wall temperatures has been proposed to enable further cloud experiments, such as studying the initiation of precipitation (17–22). The purpose of this work is to propose and explore a method for generating a realistic cloud–clear-air interface, with entrainment driven by the turbulent boundary layer below a temperature inversion in this future chamber. We present an approach that leverages the capabilities of this future chamber to generate a cloud top for detailed exploration of the entrainment process and its impact on cloud microphysics. To the best of the authors’ knowledge, a stable cloud to clear-air interface has never been achieved in a laboratory setting. The chamber’s design configuration, potential experiments, and expected results are analyzed using LES. This unique opportunity would significantly enhance the scientific value of building such a chamber.

## Results

### The Design of the Chamber.

**Fig. 1.**
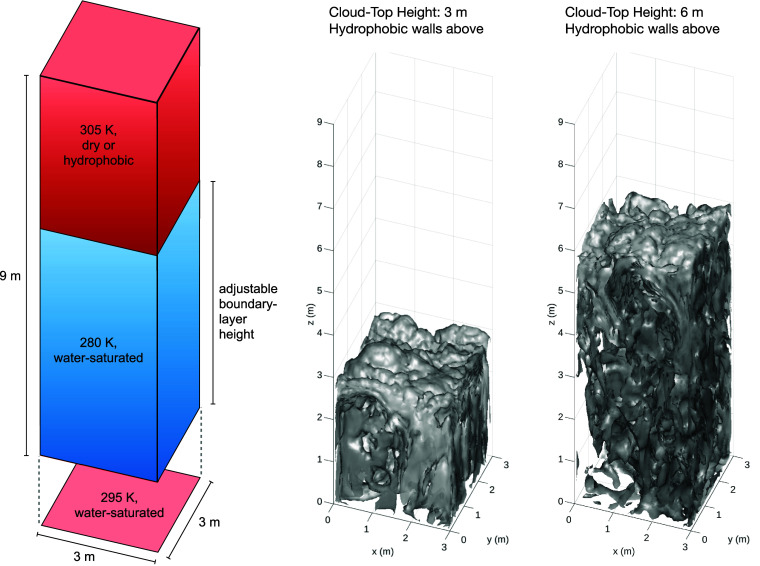
Illustration of the proposed cloud-chamber boundary conditions and two examples showing the simulated liquid water content. The presented isosurfaces display liquid water contents of chosen values (from 0.01 to 0.40 g kg^−1^). The lowest value is used to reveal the shape of the top, while the higher values are used to fill the lower part within the cloud.

The proposed convection-cloud chamber is designed with dimensions of 3m×3m×9m. A convection-cloud chamber, such as the Pi Chamber, produces Rayleigh-Bénard convection by maintaining a warm bottom surface and a cold top surface, with the sidewall temperature set between the top and bottom temperatures. However, to replicate a temperature inversion of a STBL, the upper region of the chamber needs to be warmer than the lower region. Thus, the top wall cannot serve as the cold air source for convection, requiring the sidewalls to fulfill this function. Specifically, the sidewalls should be divided into upper and lower sections: The upper section should match the temperature of the top wall, while the lower section should be colder than the bottom wall. The colder part of the sidewalls produces an influence similar to cloud-top radiative cooling by driving the mixing below the cloud top and maintaining the temperature gradient between the cloud and the cloud-free space. This temperature setup is illustrated in Fig. 1, which shows the specified temperatures for the top wall, upper sidewalls, lower sidewalls, and bottom wall as 305 K, 305 K, 280 K, and 295 K, respectively. Such a configuration facilitates convection development in the lower region of the chamber (referred to as the mixed layer hereafter), while the region above the top of the mixed layer remains stable and warmer than the lower region.

Regarding wetness of the walls, we set the bottom wall and lower sidewalls saturated with respect to liquid water. This can be achieved in the real chamber by covering them with glass fiber papers, as utilized in the Pi chamber (10), or using some hydrophilic materials (23). The upper sidewalls and top wall should ideally be as dry as possible. However, if an ideal material that keeps the walls dry while maintaining their temperature is not feasible, we also test a hydrophobic (water-repellent) condition (23). In numerical simulations, the dry condition implies that the walls always act as a moisture sink, whereas the hydrophobic condition means the walls neither evaporate nor condense liquid water, causing no moisture flux.

To assist the formation of cloud droplets, hydrophilic aerosols need to be injected to serve as cloud condensation nuclei after the turbulent convection reaches a steady state without cloud. To simplify the experiment, we inject NaCl aerosols with a constant radius of 62.5 nm into the four horizontally central grid cells at a height of 1.5 m. $1.215\times 10^{7}$ aerosols are injected into the chamber per second, which is equivalent to 0.15 cm^−3^ s^−1^ when divided by the chamber’s volume. Nevertheless, most of them will not rise beyond the mixed layer.

Since the sizes of the lower and upper sections of the sidewalls are adjustable, we can investigate the impact of cloud depth on the scientific issue of interest. For example, in this study, we test mixed-layer depths of 3 m and 6 m. The liquid water content, depicted in three-dimensional plots in Fig. 1, demonstrates that a cloud can be formed with cusps and hummocks at the cloud top, which are features created during entrainment (6, 9, 24).

### The TKE Profiles and Budgets.

**Fig. 2.**
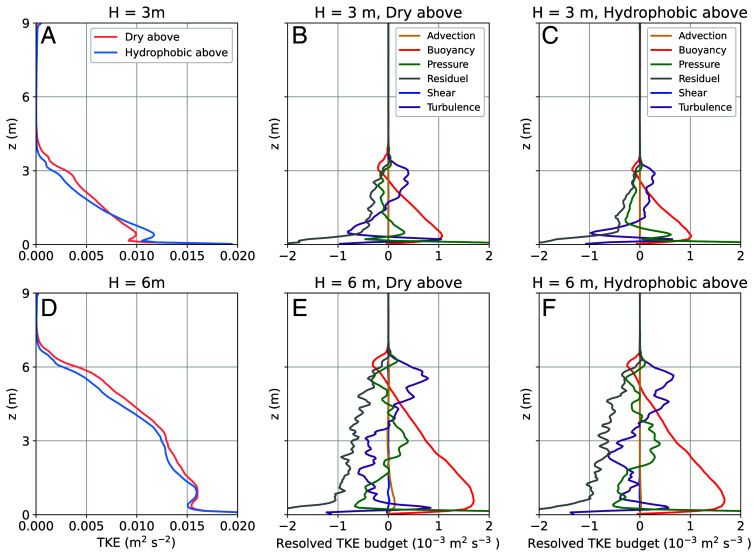
The resulting TKE and TKE budget for conditions where the mixed layer depth is 3 m (*A*–*C*) or 6 m (*D*–*F*). The TKE budgets show the terms of advection (yellow), buoyant production (red), pressure correlation (green), residual that roughly represents the dissipation of resolved TKE (gray), shear production (blue), and turbulent transport (purple).

As one of the most important variables in the STBL, turbulent kinetic energy (TKE) directly affects the fluxes of momentum, heat, moisture, and particles. Fig. 2 presents the TKE resolved by simulations and the physical processes that contribute to its generation (i.e., the TKE budget). Fig. 2*A* and *D* show that the TKE peaks are located below the middle of the mixed layer. When the mixed-layer depth is 3 m, TKE decreases more rapidly with height. A TKE peak located below the middle of the mixed layer has also been observed in previous studies (25–28), while TKE profiles can be more uniform within the mixed layer under increased instability (29).

The TKE budget in Fig. 2 reveals that the buoyancy term has the smoothest profile, indicating statistical robustness. As the main source of TKE, the buoyancy term is highest near the bottom, decreases with height, and eventually becomes a sink at the top of the mixed layer due to the presence of a temperature inversion. This profile aligns with observations in convective boundary layers (e.g., refs. 7, 27, 30, and 31), but it lacks the feature of top–down driven mixing from stratocumulus clouds (e.g., refs. 32 and 33). This is because cloud-top cooling is small compared to the heating from the bottom under this wall-temperature setting. The shear production and advection terms are negligible due to the limited horizontal extent of this tall chamber. The turbulent transport term is negative in the lower half and positive in the upper half, representing the upward transport of buoyancy-generated TKE. The pressure correlation term is out of phase with the turbulent transport term. The dissipation term, approximated by the residual in our simulation, acts as a sink and is most intense near the bottom.

Compared to the real STBL, a main difference in the proposed cloud chamber is the absence of a cloud base (Fig. 1), as well as the associated features of the TKE budget at that level. This results from the two different mechanisms for cloud formation: mixing in the chamber versus ascent and adiabatic cooling in real stratocumulus clouds. Since the purpose of this experiment is to explore cloud-top entrainment, the absence of a cloud base is acceptable.

### The Flow and Microphysical Profiles.

**Fig. 3.**
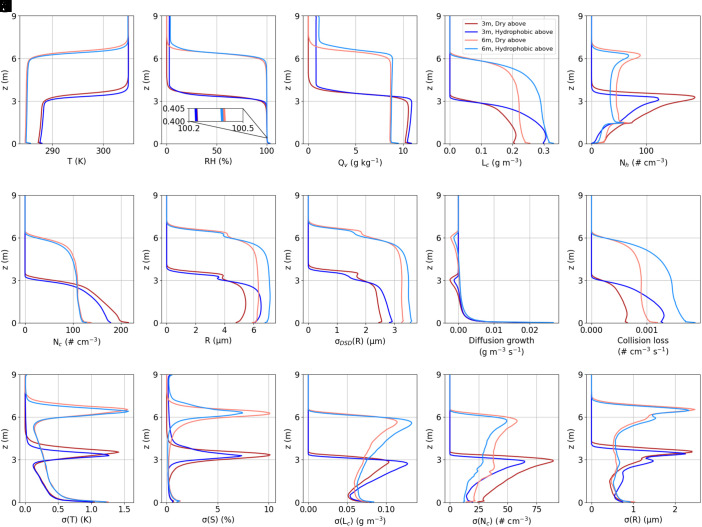
The temporal and horizontal averages of the vertical profiles for (*A*) temperature, (*B*) relative humidity (including a zoomed-in area to highlight the supersaturation), (*C*) water vapor mixing ratio, (*D*) liquid water content, (*E*) haze number concentration, (*F*) droplet number concentration, (*G*) mean droplet radius, (*H*) SD of the droplet size distribution, (*I*) diffusional growth of droplets, (*J*) collision loss of droplets, and the horizontal spatial SD of (*K*) temperature, (*L*) supersaturation, (*M*) liquid water content, (*N*) droplet number concentration, and (*O*) droplet radius. Note that the difference between panels (*H* and *O*) is that the former shows the SD of the horizontally averaged droplet size distribution, whereas the latter shows the horizontal SD of the mean radius at each grid point.

The vertical profiles of flow and microphysical properties are shown in Fig. 3. The entrainment interface layer (EIL), which separates the boundary layer and free atmosphere, is shown to be roughly 1 m, whereas the EIL in the real atmosphere is 10 to 100 m (e.g., refs. 34 and 35). The turbulence velocity scale in the cloud chamber is on the order of 0.1 m s^−1^ (estimated from the TKE profile, as shown in Fig. 2), whereas that in the convective PBL is on the order of 1 m s^−1^ (29). Thus, the time scale for mixing to penetrate the EIL is comparable in both the cloud chamber and the convective PBL.

Examining the profile of each quantity, a sharp temperature gradient can be observed at the cloud top (Fig. 3*A*). The relative humidity is also characterized by a strong gradient at the cloud top (Fig. 3*B*), but the hydrophobic upper walls allow for additional moisture above the cloud top because of detrainment (Fig. 3*B* and *C*). This addition of moisture beyond the cloud top is more evident when the cloud top is higher (cf. the light blue lines to the dark blue lines in Fig. 3*B* and *C*), due to the larger TKE and moisture within the mixed layer. However, the hydrophobic upper walls can produce a higher liquid water content (Fig. 3*D*) because of reduced evaporation near the cloud top (Fig. 3*I*). The evaporation at the cloud top turns cloud droplets to haze (inactivated aerosols), causing a peak in haze number concentration there (Fig. 3*E*). Note that another local peak of haze at 1.5 m high is where the aerosol is injected. The number concentrations of both cloud droplets and haze are less in deeper clouds (Fig. 3*E* and *F*), primarily due to the lower injection rate over the mixed layer (note that the point injection rate is the same for deep and shallow clouds over the entire domain). A minor peak in droplet radius and its distribution width is observed above the cloud top (Fig. 3*G* and *H*), possibly due to the faster evaporation of small droplets, leaving larger droplets surviving (36). Diffusion growth of droplets is negative near the cloud top due to entrainment and the most positive near the bottom because of the highest supersaturation there (cf. Fig. 3*B* and *I*). Collision–coalescence is the strongest near the bottom and decreases with height, largely following the pattern of liquid water content (cf. Fig. 3*D* and *J*). Regarding the horizontal variability, the fluctuations in temperature and supersaturation are both strongest near the cloud top due to entrainment and detrainment processes occurring there (Fig. 3*K* and *L*), consistent with previous LES studies of the STBL, which show maximum fluctuations of liquid water static energy above the mixed-layer top (37). Affected by entrainment, the horizontal variability of the microphysical properties is also highest near the cloud top (Fig. 3*M*–*O*). The relative SD for the microphysical properties ranges from 0.1 to 0.5 farther than 1 m below the cloud top (*SI Appendix*, Fig. S3), comparable to flight observations (38). Near the cloud top, the values can be extremely high as the liquid water content and number concentration approach zero.

When comparing the dry and hydrophobic upper walls (indicated by the red and blue lines in Fig. 3, respectively), stronger evaporation near the dry upper walls causes the droplet radius to decrease (Fig. 3*G*), thereby reducing fall-out removal and increasing the number concentration (Fig. 3*F*). Above the mixed layer, the width of the droplet size distribution is greater with the dry upper walls because stronger evaporation leads to more intense fluctuations in supersaturation (Fig. 3*H* and *L*). In contrast, within the cloud, the width is greater with the hydrophobic upper walls due to the overall larger droplets (Fig. 3*G* and *H*). Collision–coalescence is more frequent with hydrophobic upper walls, as it is positively correlated with liquid water content (39).

The sensitivity tests with various configurations show that a mixed-layer depth of 6 m produces greater TKE and cloud liquid water content compared to the case with a 3-m mixed-layer depth. Both dry and hydrophobic upper walls can lead to observable entrainment-induced evaporation. Since hydrophobic walls are more feasible with practical materials, we use a 6-m mixed layer with hydrophobic upper walls in the following analysis to explore the influence of entrainment on cloud microphysics.

### Inhomogeneous Mixing and Entrainment Rate.

**Fig. 4.**
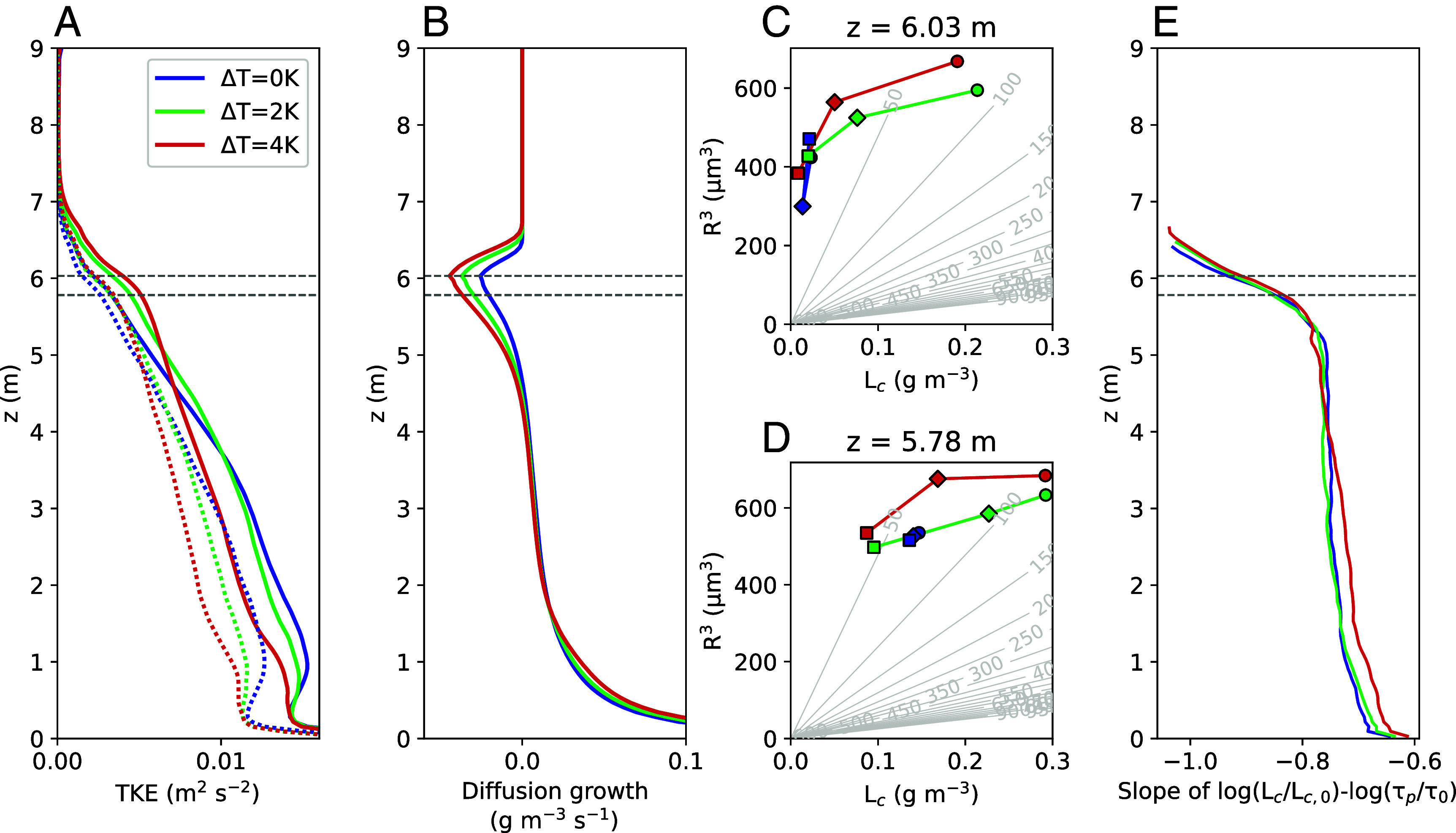
Vertical profiles of (*A*) TKE (dotted lines represent dry simulations), (*B*) diffusion growth, and (*C* and *D*) the radius cube versus liquid water content measured at two heights (indicated by dashed lines in the other panels) at different horizontal locations (circle markers: upwind; diamond markers: center; square markers: downwind). Gray lines in the background of panels (*C* and *D*) shows droplet number concentration calculated from the mean radius and liquid water content. Panel (*E*) shows the slope of the log(L*_c_*/L_*c*,0_)-log($\tau_{p}$/$\tau_{0}$) plot against height (shown for $L_{c}>10^{-4}$ g m^−3^). Colors distinguish different LSC intensities (implemented via ΔT applied to the sidewalls).

The first approach to investigating entrainment mixing types (homogeneous or inhomogeneous) is to follow Yeom et al.: measuring the droplet size distribution following an air parcel during its mixing process with subsaturated air (14). The key difference is that Yeom et al. (14) manually pump warm, subsaturated air into the Pi Chamber, whereas here, entrainment occurs through turbulent mixing at the cloud top. The large-scale circulation (LSC) is controlled by implementing a temperature difference (ΔT) across the sidewalls (see *Materials and Methods* for details). The presence of the cloud enhances the TKE of the flow (cf. dotted versus solid lines in Fig. 4*A*), and increased ΔT further strengthens TKE and cloud-top evaporation (Fig. 4*A* and *B*).

Inhomogeneous mixing is reflected in the fact that the decrease in liquid water content occurs due to the reduction in droplet number concentration rather than a reduction in droplet radius (14, 15, 40). When ΔT is applied and the upwind-downwind direction is fixed, results show mostly inhomogeneous mixing near the cloud top (Fig. 4*C* and *D*). However, when the height of the sampled data is too close to the cloud top, the droplet radius evidently decreases when liquid water content is too low (Fig. 4*C*). This is because the downwind droplets are almost entirely evaporated there, including the large ones (*SI Appendix*, Fig. S3), but a few haze particles may occasionally be activated, thus reducing the mean radius.

The second approach to examining inhomogeneous mixing is using liquid water content ($L_{c}$) and phase relaxation time ($\tau_{p}$). $\tau_{p}$ measures the time required to restore saturation in an entrainment-affected parcel (41, see Eq. **2** in *Materials and Methods*). In short, Yeom et al. shows that $\tau_{p}\propto L_{c}^{-1}$ in inhomogeneous mixing and $\tau_{p}\propto L_{c}^{-1/3}$ in homogeneous mixing (38). Thus, the slope of their relationship in logarithmic scales can be used to represent the mixing signature. Fig. 4*E* shows that, regardless of the intensity of the LSC, the mixing appears to become increasingly inhomogeneous with height, with the most significant increase occurring near the cloud top. This trend is consistent with many observations in stratocumulus clouds (38, 42, 43). However, it is often interpreted as a result of differences in cloud base heights and liquid water content that the aircraft fly through (42). In the tall cloud chamber, there is limited horizontal extent and no variation in cloud base height. Additionally, the simulation without side-wall ΔT exhibits a similar degree of inhomogeneous mixing as the other cases. Compared to the previous approach that requires measurements at upwind and downwind locations, the benefit of this approach is that the mixing signature can be examined without controlling the LSC direction.

Entrainment rate ($w_{e}$) in the atmosphere partially determines the growth rate of the STBL depth. In the proposed cloud chamber, the cloud depth is steadily maintained by the wall temperatures, so $w_{e}$ cannot be estimated from the growth rate of the cloud depth. However, the influence of entrainment can be observed in the microphysical response of the cloud droplets, indicating that entrainment is indeed occurring. This means that $w_{e}$ must be balanced by the detrainment rate. Thus, we employ the flux budget equations with dry simulations to estimate $w_{e}$ (see *Materials and Methods* for details), and Table 1 compares the estimated $w_{e}$ with the vertical velocity fluctuation at the mixed-layer top (${\sqrt{\bar{w^{\prime 2}}}}_{z=6\;m}$). Overall, enhanced LSC always increases $w_{e}$. Estimating $w_{e}$ by the mean temperature above the mixed-layer top (i.e., $T_{e}=T_{\mathrm{stable}}$) results in a scale of 0.1 mm s^−1^, but a large portion above the mixed-layer top may remain stratified and unmixed with the mixed layer. Alternatively, using the temperature at the mixed-layer top (i.e., $T_{e}=T_{z=6\;m}$), $w_{e}$ has a scale of several mm s^−1^, which agrees with some field measurements [e.g., the Second Dynamics and Chemistry of Marine Stratocumulus field study, DYCOMS-II (44, 45).] In another numerical setting that artificially places passive tracers without losses through the walls, we also found that the entrainment rate of the dry convection has a scale of several millimeters per second (*SI Appendix*, Fig. S4). Nevertheless, the estimated $w_{e}$ accounts for only roughly 10% of the vertical velocity fluctuation. This ratio was found to be 2.5% to 5% in the experiments by Deardorff and Willis (8). This suggests that most of the vertical velocity fluctuation is due to gravity waves rather than effective entrainment. In a real STBL where wind shear is stronger, the Helmholtz instability may be more pronounced, and a higher proportion of the vertical velocity fluctuation may contribute to entrainment.

**Table 1.** A comparison of the estimated entrainment rate by the flux budget equation and vertical velocity fluctuation at the mixed layer top in the dry simulations (the dotted lines in Fig. 4)

Case	$w_{e}$ $\left(T_{e}={\overset{¯}{T}}_{\mathrm{stable}}\right)$	$w_{e}$ $\left(T_{e}=T_{z=6\;m}\right)$	${\sqrt{\bar{w^{\prime 2}}}}_{z=6\;m}$
ΔT=0 K	0.197 mm s^−1^	2.97 mm s^−1^	29.7 mm s^−1^
ΔT=2 K	0.257 mm s^−1^	3.80 mm s^−1^	38.3 mm s^−1^
ΔT=4 K	0.324 mm s^−1^	4.86 mm s^−1^	41.1 mm s^−1^

### Estimation of Cloud-Wall Radiation Effect.

Although cloud-top radiative cooling plays an important role in the real atmosphere, this process is considered of secondary importance in the cloud chamber and is not modeled in the current LES setup. Unlike real stratocumulus clouds, where radiation can be considered one-dimensional, the sidewalls result in three-dimensional radiative effects. Though a three-dimensional radiation scheme is not available in the LES for this study, the influence of radiation between the cloud and sidewalls can be estimated using a Monte Carlo method based on the LES output. Detailed calculations and corresponding values are provided in *SI Appendix*.

The radiative heating rate profile indicates that the net long-wave radiative effect heats the bottom and top of the cloud while cooling the middle. This is different from a canonical profile in stratocumulus, which shows localized cooling near the cloud top and a deeper layer of weaker long-wave heating in the lower part of the cloud (24, 46). Although the absolute magnitudes of the heating and cooling rates in the chamber can be comparable to those in stratocumulus clouds, their relative importance in the chamber is small compared to the sensible heat flux from the walls and vertical transport (*SI Appendix*, Fig. S6*B*). Specifically, the net radiative heating is 3.6 K h^−1^ near the bottom, −3.8 K h^−1^ in the middle, and 2.0 K h^−1^ near the cloud top. In contrast, the sensible heat flux from the sidewalls has a peak of −47 K h^−1^, which is balanced by vertical sensible heat flux and latent heat exchange. The sidewall sensible heat flux decreases with height from its peak near the bottom, but at the cloud top, this flux combined with evaporative cooling still results in −22 K h^−1^. Thus, if three-dimensional radiation were included in the simulation, it would cause a minor change in cloud temperature, and the profiles of sensible heat flux and latent heat release/absorption would be slightly adjusted to balance the radiative heating.

In summary, compared to the real atmosphere, the amount of liquid water in the cloud chamber is relatively transparent to long-wave radiation. With the current wall-temperature setup, our estimation shows that the radiative effect is negligible. However, it is possible that a different wall-temperature setup may result in noticeable radiative effects, and this should always be checked when configuring the wall temperatures.

## Conclusion and Discussion

Using LES, we demonstrate how a stable cloud-top interface can be created in a tall convection-cloud chamber under well-controlled wall conditions. The turbulent cloud is driven by saturated cold sidewalls and a warm bottom wall, while a temperature inversion is maintained by the upper sidewalls and a top wall that are warmer than the bottom. The TKE profiles and budgets resemble those observed in the convective boundary layer in many aspects. The vertical profiles reveal several features that can be explored at the cloud top, such as increased haze number concentration, droplet size variability, and fluctuations in temperature and supersaturation. The configurations also allow for the investigation of entrainment signature and rate. LES results show that the microphysical response to entrainment becomes increasingly inhomogeneous with height in the chamber, consistent with observations in the STBL (38). Additionally, a flux-budget model can be combined with measured wall fluxes to estimate the entrainment rate. Last, although the estimation of the radiative effect is small compared to the sensible heat fluxes in the current model setup, it needs to be carefully assessed when using a different wall-temperature setup. Compared to measurements in the STBL, the cloud chamber provides controllable boundary conditions, a steady-state cloud, and more detailed measurements. These advantages will improve our understanding of cloud-top entrainment and offer insights for microphysical modeling in atmospheric simulations that cannot be achieved in other natural or laboratory settings.

One question concerning the construction of the proposed chamber is: “In a real chamber, can we implement the two types of boundary conditions for moisture above the cloudy layer assumed in the LES?” In short, moisture-absorbing walls are technically challenging, whereas moisture-adiabatic walls are feasible. Moisture-absorbing walls require the ability to absorb water (e.g., via a desiccant) without becoming saturated, whereas moisture-adiabatic walls create no driving force for condensation, as the relative humidity at the walls remains below 100%. Therefore, most standard wall materials should suffice for moisture-adiabatic walls.

In contrast to turbulence, which tends to uniformly distribute the cloud, sedimentation of droplets away from the cloud top may also affect the entrainment rate, and this influence can be explored in the proposed setting. Ackerman et al. and Bretherton et al. found, using LES of stratocumulus-topped boundary layers, that droplet sedimentation near the cloud top reduces the liquid water available to evaporatively cool entrained air and thereby reduces the entrainment rate (47, 48). A recent study using meter-scale DNS of a stratocumulus-topped boundary layer supports these conclusions and additionally finds that the impact of sedimentation on entrainment rate increases with Reynolds number (49). The latter study suggests that microphysical effects, including sedimentation, are equally important as turbulence effects in determining entrainment, and that meter-scale resolution is needed to correctly represent these effects.

Compared to traditional cloud chambers, which are filled with cloud particles, the cloudless region offers greater flexibility for placing instruments. For example, if a drier region above the cloud top is desired and dry walls are not practical, a dehumidifier can be placed there. If an additional aerosol/haze sink is needed to maintain the steadiness of aerosol/haze numbers above the cloud, an air purifier can be used. Additionally, while installing a fan in traditional cloud chambers is typically considered a desirable way to enhance turbulence, it can capture, break, and spray cloud droplets, thereby disturbing the clouds. However, these concerns do not apply when installing a fan to drive the LSC above the cloud top, which significantly broadens the range of possible experiments, e.g., including the wind shear effects. Finally, the space can be used to test remote sensors such as high-resolution lidar (50, 51) and radar (52).

**Fig. 5.**
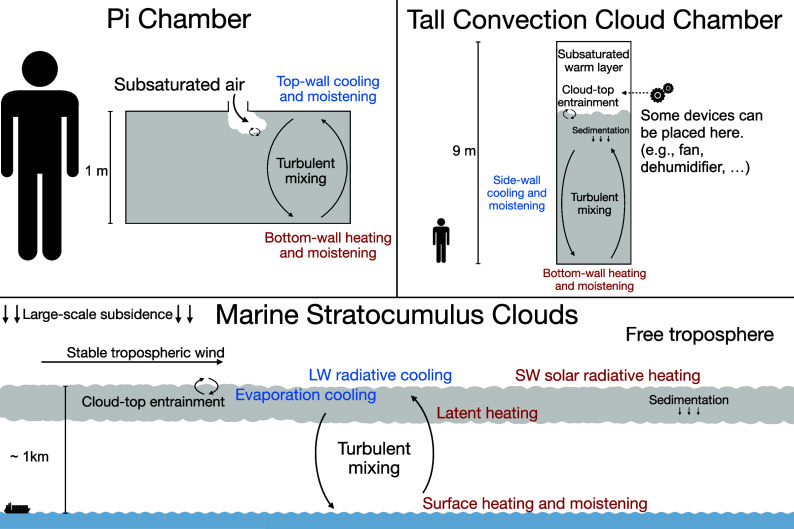
A schematic comparing the mechanisms driving turbulence and entrainment in the Pi Chamber (14, 15), the large-scale chamber (explored in this study), and the marine stratocumulus clouds. Red and blue fonts indicate the dominant warming and cooling sources, respectively.

One of the purposes of a cloud chamber is to provide well-defined conditions and isolate specific processes. Fig. 5 compares the entrainment that occurs in the Pi Chamber, the tall convection-cloud chamber proposed and explored in this work, and in marine stratocumulus clouds. Compared to the Pi Chamber, the large-scale chamber provides a more natural cloud top with a TKE profile closer to that of a convective boundary layer. The deeper cloud provides sufficient space to explore how the influence of entrainment varies with height, as well as enough depth to investigate the possible influence of sedimentation, while the upper dry area offers room for additional instruments. Compared to marine stratocumulus clouds, the large-scale chamber lacks a cloud base, radiative effects, subsidence, externally imposed vertical wind shear, and horizontal variability in properties caused by organized mixed-layer circulations.

An open challenge is to understand how the length and time scales in the simulated laboratory experiment translate to an atmospheric context. For example, the transition from strongly inhomogeneous mixing at the cloud top to more homogeneous-like mixing deeper in the cloud has been observed in stratocumulus clouds (38, 43), but it occurs on scales of tens to hundreds of meters. In the simulation, however, the transition occurs on a meter scale. Is the length relative to the cloud depth the relevant quantity, in which case the scaling is straightforward, or is there other physics at play? The approach to the laboratory experiments simulated here has been to match the microphysical time scales, such as growth/evaporation rates and phase relaxation time. Turbulent mixing occurs, but the largest spatial and temporal scales present in the atmosphere are not matched. As a result, processes directly influenced by the largest scales are not quantitatively reproduced, whereas processes that depend on inertial range scales corresponding to microphysical scales (e.g., ref. 40) should be simulated properly.

Finally, there are more topics to explore with this configuration, such as the investigation of cloud-droplet settling effects near the cloud–clear-air boundary and their influence on entrainment (48). The flexible wall-temperature setup offers additional possibilities; by reducing the bottom wall’s heating and implementing a negative temperature gradient on the sidewalls, with the coolest temperatures at the cloud-top height, we may be able to explore a top–down driven boundary layer similar to a real stratocumulus-topped boundary layer (e.g., refs. 32 and 33). Beyond steady states, the wall temperature could vary with time. Specifically, boundary layer development could be examined by initially setting the sidewall temperature to increase with height and gradually raising the bottom-wall temperature. The resulting buoyancy increase would generate thermal bubbles that penetrate the mixed-layer top, mixing warmer air into the boundary layer and increasing its height. When the sidewalls are saturated, the rising bottom-wall temperature would gradually increase supersaturation, leading to cloud formation. Looking further ahead, experiments could provide insights into whether evaporative cooling might make a mixed air parcel cooler than the cloud, triggering entrainment instability and breaking up the cloud (1, 48, 53). The results explored through LES and other potential experiments all demonstrate the value of this future laboratory facility.

## Materials and Methods

### LES with Bin Microphysics Scheme.

The simulations are performed using the System For Atmospheric Modeling (SAM) LES (54), which has been utilized in numerous cloud-chamber simulations (13, 15, 17–19, 22, 55–59). The grid spacing is 6.25 cm within a 3 m by 3 m by 9 m cuboid domain, resulting in 48 by 48 by 144 grid cells in each direction. This grid spacing falls within the inertial subrange of similar cloud-chamber flows examined by direct numerical simulation (60), justifying its use for the LES here. The subgrid-scale turbulent fluxes are modeled by the TKE scheme (61). The wall fluxes are modeled based on Monin–Obukhov similarity theory (62), with the parameters tailored for the cloud chamber as detailed in ref. 18. The bin microphysics model simulates droplets of each size bin using the haze-capable Chen-and-Lamb scheme detailed in ref. 56. A wall-loss time scale of 10 min is applied to aerosol and haze, as per refs. 18 and 55.

The simulations run for 180 min. The first 30 min are used to spin up the moist turbulent thermal convection. Starting from t=30 min, aerosols are continuously injected from the central four grid points at z=1.5 m. Most flow and microphysical properties reach a steady state before t=90 min (*SI Appendix*, Fig. S1), and the results from t=120 to 180 min are averaged for examination. Some properties above the cloud top change slightly over time, as the air there is less turbulent than the convection within the cloud. Nevertheless, analysis indicates that the time scales for these changes to affect the cloud region are much longer than the analyzed time, on the order of 1 h (*SI Appendix*, Fig. S2 and Table S1).

An additional set of dry simulations (without aerosols or moisture) is performed to examine the influence of clouds on the TKE of the LSC (results are shown as dotted lines in Fig. 4). Without clouds, the convection reaches a steady state much faster, and thus only 120 min of simulation is carried out, with t=60 to 120 min used for analysis.

### Calculation of TKE Budget.

Analyses of TKE budget are calculated from the resolved quantities in LES. Here, the subgrid-scale TKE and its budget are not considered. The calculation mainly follows equation (5.1a) in ref. 30. Specifically,

[1] $$\begin{array}{c} \frac{\partial \bar{e}}{\partial t}={\overset{¯}{u}}_{j}\frac{\partial \bar{e}}{\partial x_{j}}+\delta_{i3}\frac{g}{{\overset{¯}{T}}_{v}}\left(\bar{u_{i}^{\prime }T_{v}^{\prime }}\right)-\bar{u_{i}^{\prime }u_{j}^{\prime }}\frac{\partial {\overset{¯}{u}}_{i}}{\partial x_{j}}-\frac{\partial \left(\bar{u_{j}^{\prime }e}\right)}{\partial x_{j}}-\frac{1}{\overset{¯}{\rho }}\frac{\partial \left(\bar{u_{i}^{\prime }p^{\prime }}\right)}{\partial x_{i}}-\varepsilon , \end{array}$$

where e is TKE, $u_{i}$ is velocity, δ is the Kronecker delta, g is gravitational acceleration, $T_{v}$ is virtual temperature (which has negligible difference from virtual potential temperature in this small domain), p is pressure, and ε is the dissipation rate. The subscripts i and j are Einstein notation. The overbar denotes the Reynolds average, which is computed here as a temporal-horizontal average. The superscript prime indicates the perturbation from the Reynolds average. The three-dimensional fields are saved every 2 min, and the Reynolds-averaged fields are obtained by averaging over t=1 to 3 h and the two horizontal directions. In this work, the dissipation rate is approximated by the residual of the other terms because $\partial \bar{e}/\partial t≅0$. The terms on the right-hand side, in order, are advection, buoyancy, shear production, turbulent transport, pressure correlation, and dissipation, respectively.

### Controlling LSC by the Side-Wall Temperature Difference.

The convection below the temperature inversion generates an LSC that is as deep as the cloud. Without horizontal variation, the direction of LSC changes over time in an idealized Rayleigh-Bénard convection (18, 63). To control the direction and intensity of LSC, we can implement ΔT across the sidewalls to maintain the direction of the LSC (64). For instance, ΔT of 2 K indicates an increase of 1 K on the left and front walls and a decrease of 1 K on the right and back walls. Note that this ΔT is applied to both the upper and lower parts of the sidewalls, meaning that increasing ΔT results in enhanced LSC with more pronounced shear at the cloud top. Fig. 4*A* shows that enhanced LSC increases TKE near the cloud top and decreases it within roughly two-thirds of the cloud volume.

### Examination of Inhomogeneous Mixing.

For the first approach to examine inhomogeneous mixing (Fig. 4*C* and *D*), the upwind and downwind results are taken from horizontally positioned 2 by 2 grid cells centered 35.4 cm away from the horizontal domain center, either upwind or downwind along the LSC. These “virtual sensors” are placed similarly to the real ones used in the Pi Chamber (14, 15).

For the second approach to examine inhomogeneous mixing (Fig. 4*E*), the scatter plot of log($L_{c}$/$L_{c,0}$) against log($\tau_{p}$/$\tau_{0}$) is used to derive the slope (i.e., the slope of the linear-fitted line). Note that $\tau_{0}$ and $L_{c,0}$ are unit values having the dimensions of $\tau_{p}$ and $L_{c}$, respectively, that are used solely to compose nondimensional ratios for computing the logarithms. Here, $L_{c}$ and $\tau_{p}$ are calculated at each individual grid point. $L_{c}$ is defined as the sum of the mass from each bin in the microphysics scheme (excluding the haze component). The calculation of $\tau_{p}$ follows ref. 38:

[2] $$\begin{array}{c} \tau_{p}=\frac{1}{4\pi D_{v}N_{c}R}, \end{array}$$

where $D_{v}$ is the modified vapor diffusivity (65).

### Flux Budget Equation for the Estimation of Entrainment Rate.

To estimate the entrainment rate, we apply the bulk scalar flux model (13, 14, 18, 55). Also, to reduce uncertainty associated with latent heat effects, we use the dry simulations for estimation (the dotted lines shown in Fig. 4*A*). The flux budget equation of temperature can be written as

[3] $$\begin{array}{c} \rho c_{p}V\frac{d\overset{¯}{T}}{dt}=\rho c_{p}A_{b}F_{b}+\rho c_{p}A_{s}F_{s}+\rho c_{p}A_{b}\left(w_{e}T_{e}-w_{d}\overset{¯}{T}\right), \end{array}$$

where ρ is the density of air, $c_{p}$ is the heat capacity of air, V is the volume of the mixed layer (54 m^3^), $A_{b}$ is the bottom area (9 m^2^), $A_{s}$ is the side-wall area (72 m^2^ for all four sidewalls), $F_{b}$ is the wall flux at the bottom, $F_{s}$ is the wall flux on the sidewalls, $\overset{¯}{T}$ is the mean temperature of the mixed layer, $T_{e}$ is the entrained temperature, and $w_{d}$ is the detrainment rate. The values listed in parentheses are for the 6-m deep mixed layer. In a chamber with a height of less than 10 m, ρ can be assumed to be constant. For a steady state, $d\overset{¯}{T}/dt=0$. Last, an unchanged mixed layer depth implies that $w_{e}=w_{d}$. Thus, Eq. **3** can be simplified to derive the entrainment rate:

[4] $$\begin{array}{c} w_{e}=\frac{-F_{b}-\frac{A_{s}}{A_{b}}F_{s}}{T_{e}-\overset{¯}{T}}. \end{array}$$

Here, $A_{s}/A_{b}=8$, and $F_{b}$ and $F_{s}$ are obtained from the model output (in a real chamber, it would be desirable to have a method for measuring the heat flux). Regarding $T_{e}$, the most intuitive option is the mean temperature of the stable layer. However, it is unlikely that turbulence can reach the warmest part of the stable layer, so we also estimate $w_{e}$ using the temperature right at the mixed layer top (i.e., z=6 m) as $T_{e}$. Results are presented in Table 1.

## Supplementary Material

Appendix 01 (PDF)

## Data Availability

The SAM model is coded by Prof. Marat Khairoutdinov and available at his personal website (66). The output is stored on NERSC HPSS storage system (67).
