# Supplementary material for: Generating a stratocumulus-like cloud top in a convection-cloud chamber
Source: Proc Natl Acad Sci U S A. 2026 Mar 12;123(11):e2519791123. doi: 10.1073/pnas.2519791123 (PMC12994161; doi:10.1073/pnas.2519791123)
Supplement: Supplementary file 1 — Appendix 01 (PDF) [file pnas.2519791123.sapp.pdf]

Supporting Information for

Generating a Stratocumulus-Like Cloud Top in a Convection-Cloud Chamber

Aaron Wang, Fan Yang, Mikhail Ovchinnikov, Steve Krueger, and Raymond A. Shaw

Aaron Wang
E-mail: aaron.wang@pnnl.gov

This PDF file includes:

Supporting text
Figs. S1 to S7
Table S1
SI References

Supporting Information Text

Examination of Steady States. For the examination of steady states, we use simulations with a cloud top at 6 m, with either dry or hydrophobic upper parts of the side walls. Figure S1 shows the time-height plots of representative properties. The turbulent kinetic energy (TKE), temperature (T), and water vapor mixing ratio (Qv) reach a steady state of moist convection within the first 30 minutes. Then, after aerosol is injected at $t = 30$ minutes, the cloud regions reach a steady cloudy state before $t = 90$ minutes.

The cloudless region above the cloud top has minimal moisture and haze, so its properties are examined separately. Figure S2 shows the time series of Qv and number concentration of haze (Nh) averaged over $z = 6$–9 m. They both reach relatively steady state, except that Qv fluctuates slightly (Fig. S2b; note that the span of the y-axis is small).

To examine whether changes in the properties above the cloud top will affect the cloud, a time scale representing whether the cloud area can feel the property change above the cloud is calculated as follows:

$$\tau_{\text{change}} = \frac{0.1 \times \phi_{\text{cloud}}}{\frac{\partial \phi_{\text{cloudless}}}{\partial t}} \frac{V_{\text{cloud}}}{V_{\text{cloudless}}}, \quad [1]$$

where ϕ is the property of interest; ϕ_{cloud} is the property averaged over $z = 0$–6 m and $t = 90$–180 minutes; $\phi_{\text{cloudless}}$ is the property averaged over $z = 6$–9 m; and $\partial\phi_{\text{cloudless}}/\partial t$ is the tendency over $t = 90$–180 minutes, illustrated by the slope shown in Fig. S2. $V_{\text{cloud}}/V_{\text{cloudless}} = 2$ is the volume ratio of the cloud region to the cloudless region. The factor of 0.1 implies that the change above the cloud needs to affect at least 10% of the properties in the cloud region. Table S1 shows that the time scales range from one day to several weeks (and even years), all of which are much longer than the simulated and analyzed time scale of an hour.

Horizontal Relative Standard Deviation of Microphysical Properties. Profiles of the horizontal standard deviations of liquid water content, droplet number concentration, and droplet radius normalized by the corresponding means obtained from Fig. 3 are presented in Fig. S3.

Droplet Size Distribution of Virtual Sensors. The droplet size distribution (DSD) used to derive Fig. 4c–d in the manuscript is presented here as Fig. S4. Note that the color in Fig. 4c–d in the manuscript is used to distinguish different large-scale-circulation intensities, whereas the color here in Fig. S4 is used to distinguish the upwind, center, and downwind locations.

Entrainment Rate Estimated by a Virtual Tracer. To exclude the influence of wall fluxes and focus solely on the entrainment flux at the cloud top, we set a virtual tracer with a concentration fixed in time at 1 for $z > 6$ m, beginning after convection reaches a steady state ($t = 1$ hr for dry simulations and $t = 2$ hr for cloudy simulations). The change in tracer concentration (C) within the mixed layer is expressed as:

$$\frac{\mathrm{d}C}{\mathrm{d}t} = -\frac{w_e}{h}\left(C - C_{\text{top}}\right), \quad [2]$$

where w_e is the entrainment rate, $h = 6$ m is the depth of the mixed layer, and C_{top} is the fixed tracer concentration at $z > 6$ m. Let $C_{\text{top}} = 1$; by solving the equation with the initial condition $C(t_0) = 0$, where t_0 is the time when the tracers are initiated, we can derive the following:

$$C(t) = 1 - \exp\left[-\frac{w_e}{h}(t - t_0)\right], \quad [3]$$

$$w_e = -\frac{h}{t - t_0}\ln\left[1 - C(t)\right]. \quad [4]$$

Note that $(t - t_0)$ here represents the time elapsed since the tracer was introduced. Equation 3 describes the temporal evolution of tracer concentration within the mixed layer, while Equation 4 provides a method to calculate w_e. Figure S5 shows simulated time series of C averaged over the mixed layer (solid lines), with the fitted line using the mean w_e derived from Equation 4. In short, enhanced LSC intensity increases w_e, and the cloudy cases evidently have higher w_e compared to the dry cases.

Radiative Effect on Cloud Evaluated by Monte Carlo Method in a Finite Cloud Chamber. Figure S6 shows the heating rate caused by long-wave radiation between the walls and cloud (panel a) and compares it with those caused by latent heat release, sidewall sensible heat flux, and vertical sensible heat flux (panel b). Here, the latent heat release and sidewall sensible heat flux are outputs from LES, the vertical sensible heat flux is calculated as their residual, and the radiation effect is evaluated using the LES cloud properties with the Monte Carlo (MC) method, as detailed below.

The geometry of the cloud chamber has an aspect ratio (horizontal length over vertical length scales) lower than one, which is different from stratocumulus clouds, where the aspect ratio is much larger than one. Thus, the traditional one-dimensional radiative transfer flux model is not appropriate. Instead, we adopt the MC method to compute the vertical profile of radiative heating in the cloudy region below $z = 6$ m in the chamber. MC radiative transfer has been widely used for cavity and cloud-radiation problems (1, 2). Lambertian wall emission and diffuse boundary conditions follow standard radiative heat transfer theory (3). The goal is to estimate the absorption of thermal infrared radiation emitted by the chamber walls into a finite, optically thin, and weakly absorbing medium consisting of cloud droplets. The method resolves the full three-dimensional geometry of the chamber and its wall temperatures.

The cloudy region occupies a rectangular volume:

$$0 \le x \le L_x, \qquad 0 \le y \le L_y, \qquad 0 \le z \le H, \tag{5}$$

where $L_x = L_y = 3\,\mathrm{m}$ and $H = 6\,\mathrm{m}$. The absorbing medium is roughly confined to this 0–6 m layer. Above $z = H$, the gas and walls are almost isothermal at $T = 305\,\mathrm{K}$ and are therefore radiatively neutral. This region is represented by an effective top boundary at $z = H$ with temperature $T_{\mathrm{top}} = 305\,\mathrm{K}$. The remaining wall temperatures are $T_{\mathrm{bottom}} = 295\,\mathrm{K}$ and $T_{\mathrm{side}} = 280\,\mathrm{K}$.

The cloud-droplet absorption coefficient (k) is calculated as:

$$k = Q_{\mathrm{abs}} N_c \pi R^2, \tag{6}$$

where N_c is the droplet number concentration, R is the droplet radius, and Q_{abs} is the absorption efficiency of liquid droplets. Here, Q_{abs} is derived using Mie Scattering (calculated with the miepython package, 4), with refractive indices provided in Ref. (5). Figure S7 compares the resulting Q_{abs} spectrum with the spectral radiance from the warmest and coolest walls. To maximize the possible influence of radiative effects, we use the maximum Q_{abs} in the infrared range (as shown in Fig. S7) at each level, given the LES cloud properties. Note that using the wave-number-dependent Q_{abs} may further reduce the influence of radiative effects on the cloud. The resulting k profile in Fig. S6a demonstrates that ($kL \ll 1$), placing the problem in the optically thin regime. Cloud self-emission is incorporated analytically via the standard thin-limit expression $4k\sigma T^4$, as described below.

Each wall emits diffuse (Lambertian) hemispheric radiation with flux $F_i = \sigma T_i^4$, where T_i is the temperature of wall i and σ is the Stefan–Boltzmann constant. The total emitted power from wall i is:

$$\dot{Q}_i = F_i A_i, \tag{7}$$

where A_i is the wall area. The MC simulation launches N photon packets (result in Fig. S6 is obtained with $N = 10^7$), each representing equal fractions of the total radiative power:

$$\dot{Q}_{\mathrm{tot}} = \sum_i \dot{Q}_i. \tag{8}$$

The probability that a photon originates from wall i is:

$$P_i = \frac{\dot{Q}_i}{\dot{Q}_{\mathrm{tot}}}. \tag{9}$$

A photon's emission point is sampled uniformly over the chosen wall's surface, and the initial propagation direction is drawn from a cosine-weighted (Lambertian) distribution over the inward hemisphere, with probability density proportional to $\cos\theta$.

After emission, the photon travels in a straight line until it intersects a wall of the chamber. The distance to the next intersection is computed analytically by solving for the smallest positive parameter t such that $(x, y, z) + t(d_x, d_y, d_z)$ lies on one of the surfaces $x = 0$, $x = L_x$, $y = 0$, $y = L_y$, $z = 0$, or $z = H$. No scattering is included, which is consistent with the optically thin cloud microphysics.

For the purpose of computing absorption in the cloud layer, the ray segment inside $0 \le z \le H$ is decomposed into subsegments determined by intersections with a set of horizontal analysis levels. If the vertical grid consists of levels $z_0 = 0 < z_1 < \cdots < z_M = H$, then each ray segment between successive intersections contributes a path length Δs_j to exactly one layer j, identified by the midpoint height of the segment.

Because $kH \ll 1$, the fractional absorption of each photon in a segment of length Δs can be approximated by $k\Delta s$. Thus the absorbed power in layer j is

$$\dot{Q}_{\mathrm{abs},j} = \left(\frac{\dot{Q}_{\mathrm{tot}}}{N}\right) \sum_{\text{rays in layer j}} k\,\Delta s_j, \tag{10}$$

where the sum is over all ray segments crossing the layer. Dividing by the layer volume $V_j = L_x L_y (z_{j+1} - z_j)$ yields the absorbed power per unit volume:

$$\dot{q}_{\mathrm{abs}}(z_j) = \frac{\dot{Q}_{\mathrm{abs},j}}{V_j}. \tag{11}$$

The local emission of the cloud is treated analytically using the linearized optically thin expression

$$\dot{q}_{\mathrm{emit}}(z_j) = 4k\sigma T(z_j)^4. \tag{12}$$

The net radiative heating rate per unit volume is then

$$\dot{q}_{\mathrm{net}}(z_j) = \dot{q}_{\mathrm{abs}}(z_j) - \dot{q}_{\mathrm{emit}}(z_j), \tag{13}$$

and the temperature tendency is

$$\frac{\partial T}{\partial t}(z_j) = \frac{\dot{q}_{\mathrm{net}}(z_j)}{\rho c_p}. \tag{14}$$

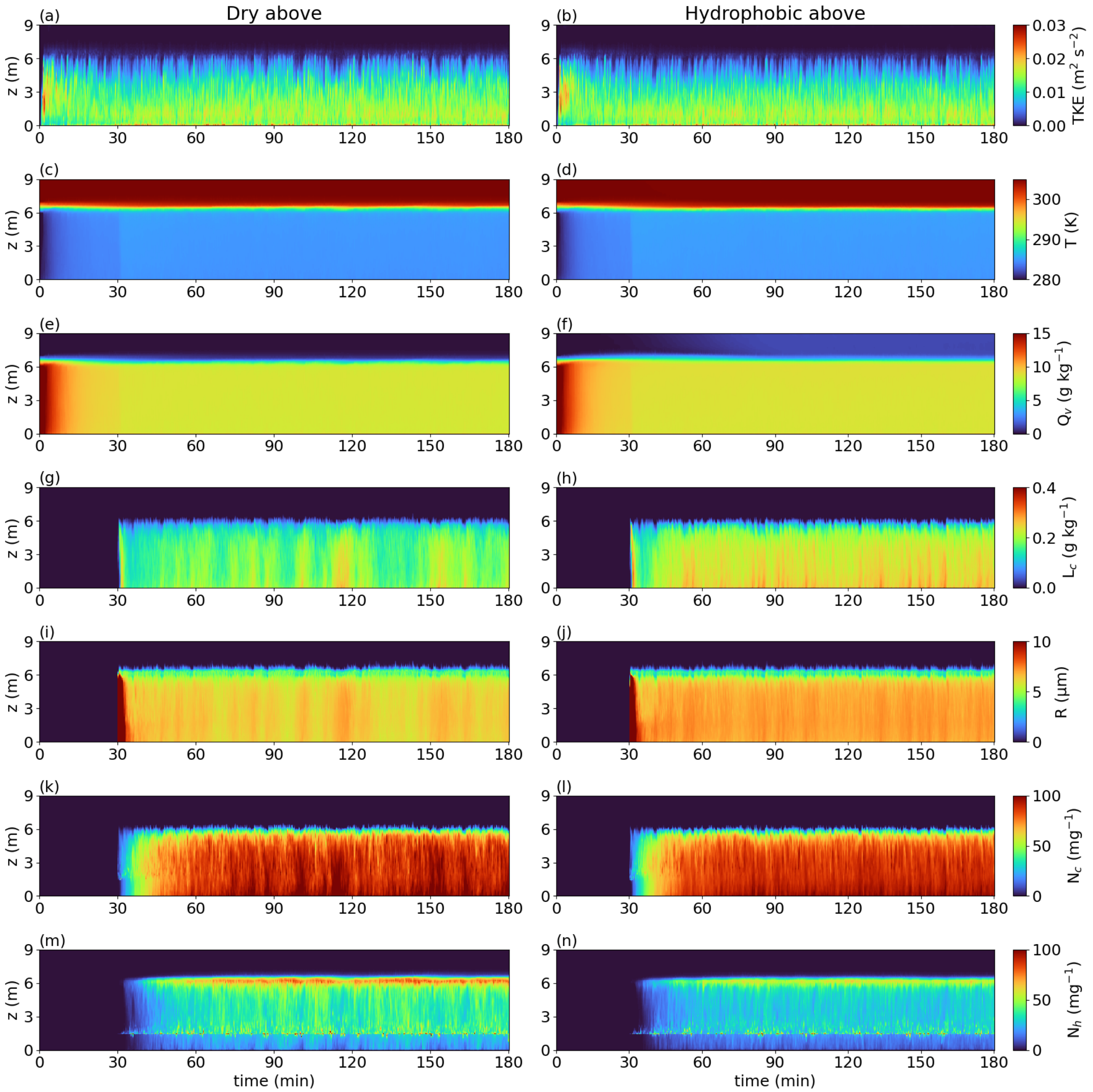

Fig. S1. Time-height plots of flow and microphysical properties (distinguished by rows) with a cloud depth of 6 m, and walls above that are either dry or hydrophobic (distinguished by columns). The rows from top to bottom display turbulent kinetic energy, temperature, mixing ratio, liquid water content, droplet radius, cloud number concentration, and haze number concentration.

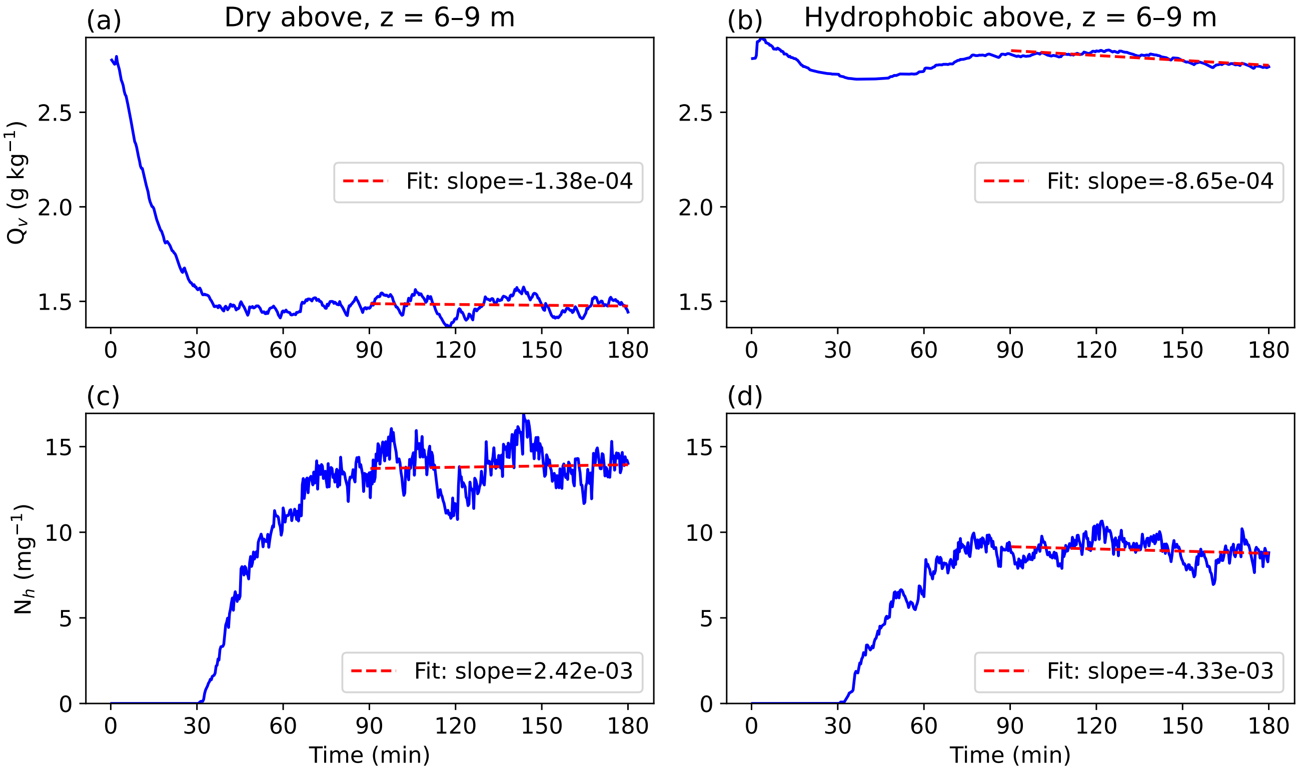

Fig. S2. The time series of (a, b) Qv and (c, d) Nh averaged over $z = 6$–9 m, with a cloud depth of 6 m and (a, c) dry or (b, d) hydrophobic walls above. The red dashed lines represent the fitted trend after t = 90 min.

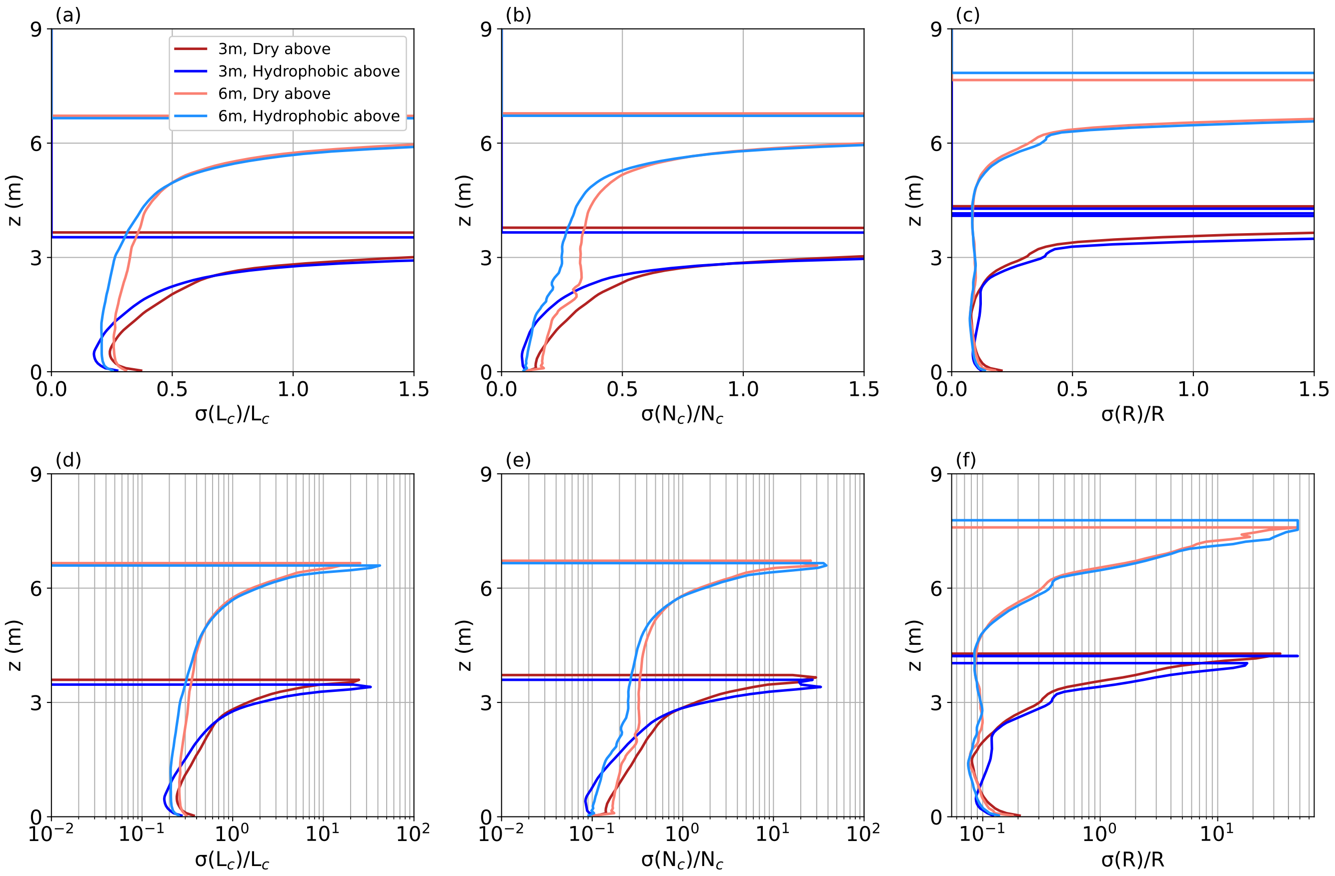

Fig. S3. The horizontal standard deviation of liquid water content, droplet number concentration, and droplet radius obtained from Fig. 3 is presented. The first row shows the linear scale for the x-axis, while the second row shows the logarithmic scale for the x-axis.

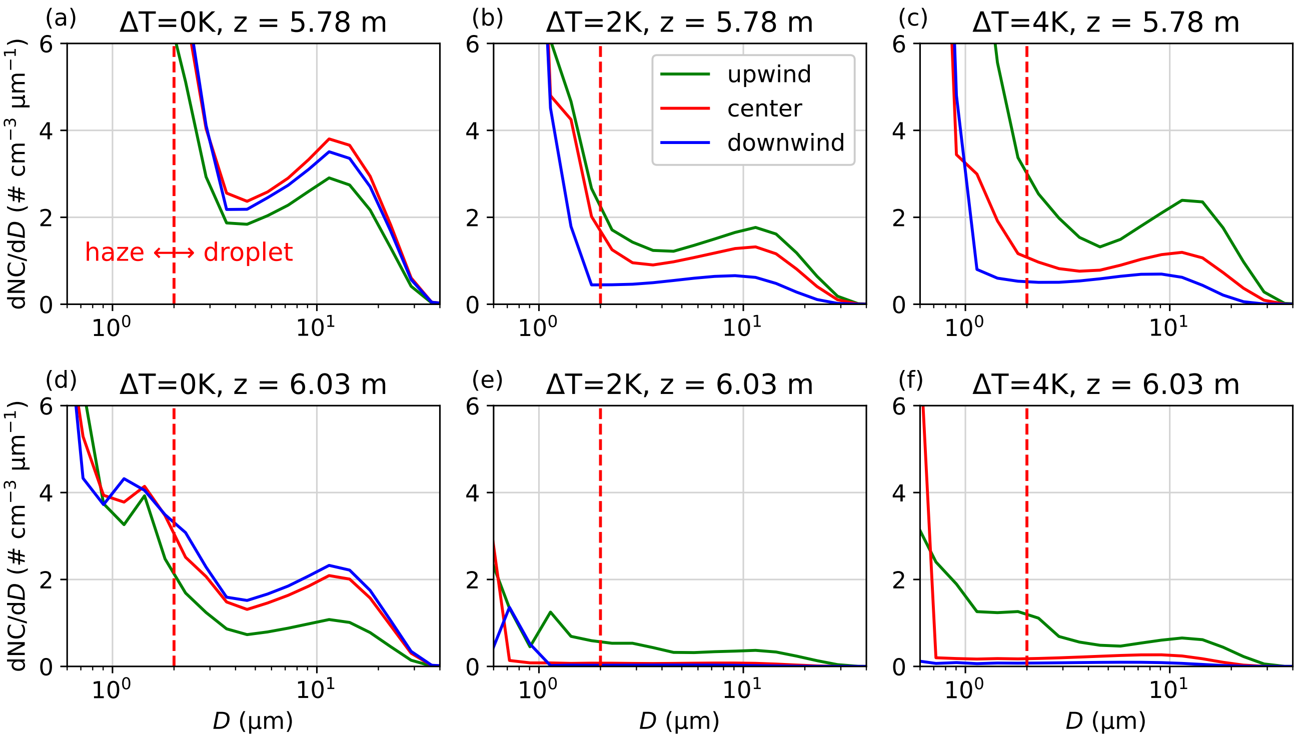

Fig. S4. The droplet size distributions at upwind (green line), center (red line), and downwind (blue line) regions at two different heights (distinguished by rows) under three different strengths of large-scale circulation (distinguished by columns).

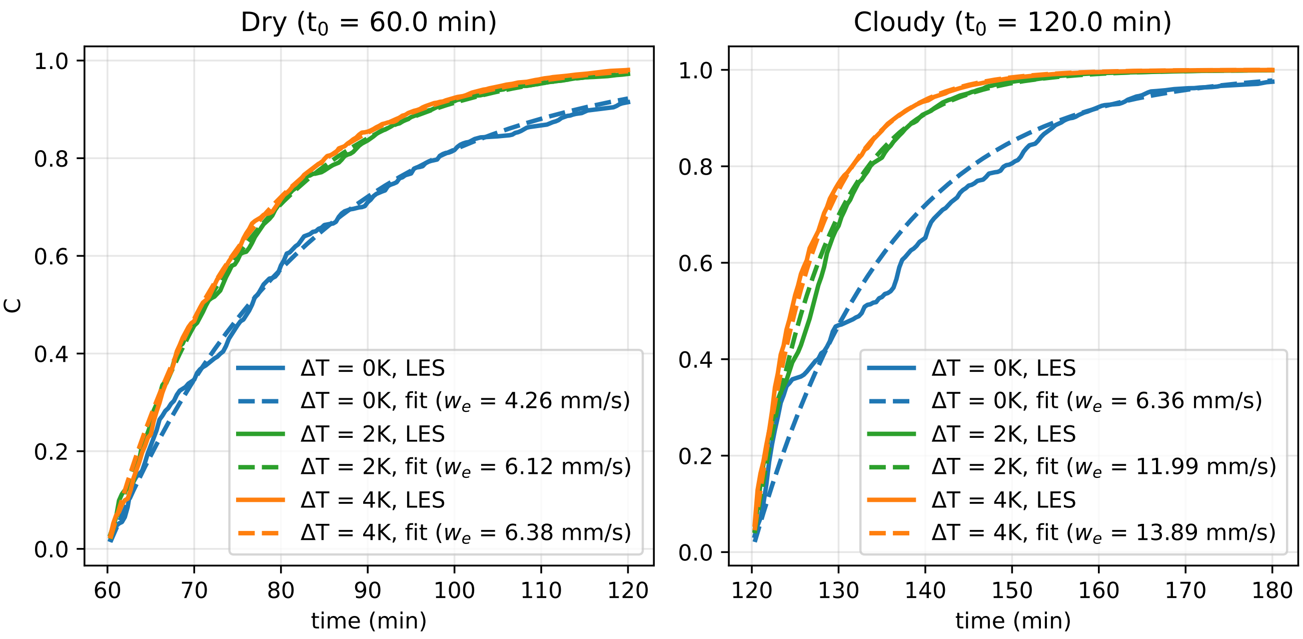

Fig. S5. The results of employing the constant passive tracer above the mixed layer. The left panel shows the dry simulations, and the right panel shows the cloudy simulations. The solid lines represent the LES results, while the dashed lines show the fitted results using Equation 3 and the entrainment rates derived from Equation 4.

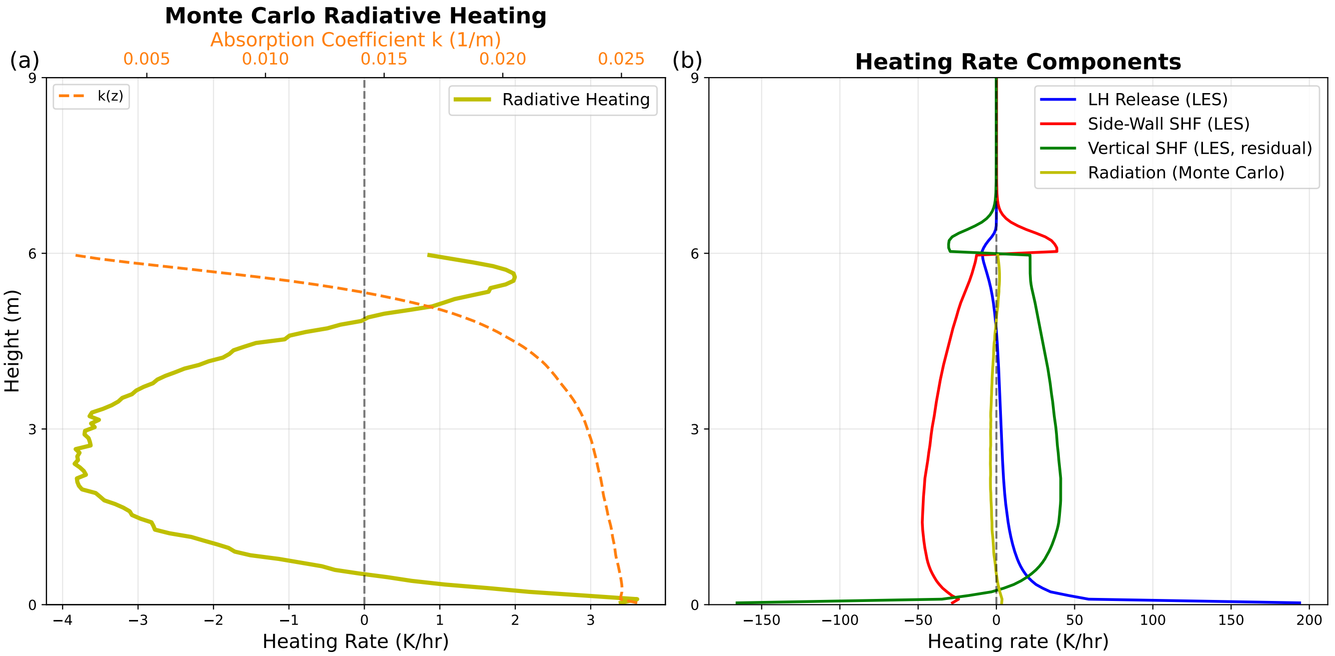

Fig. S6. The vertical profiles of (a) the radiative heating rate (yellow line) and cloud absorption coefficient (orange dashed line), and (b) the heating rate due to latent heat release (blue line), side-wall sensible heat flux (red line), vertical sensible heat flux (green line), and the radiation effect (yellow line).

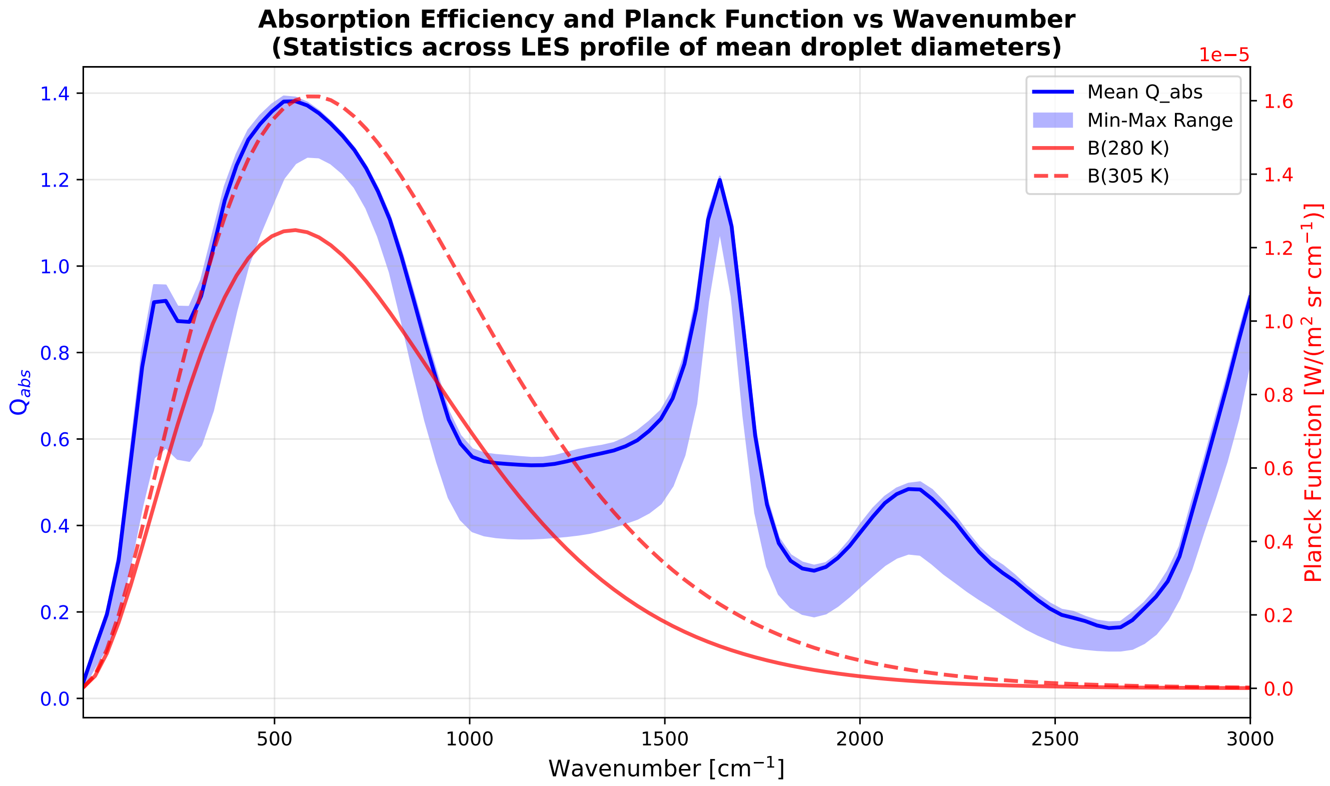

Fig. S7. The absorption efficiency (blue) for a water droplet calculated using Mie Scattering (via the miepython package, 4), the refractive index provided in Ref. (5), and N_c and R values from the LES output. Two red lines show the spectral radiance of the minimum wall flux and maximum wall flux for comparison.

Table S1. Time scales of changes in flow and microphysical properties above the cloud that affect 10% of properties within the cloud.

Property	Dry Above	Hydrophobic above
TKE	1.2 weeks	4.0 weeks
T	8.0 year	3.6 weeks
Qv	1.2 weeks	1.4 days
Lc	1.4 weeks	1.9 weeks
R	6.1 days	2.6 days
Nc	1.2 weeks	1.4 weeks
Nh	2.1 days	0.9 days

References

1. GI Marchuk, et al., *The Monte Carlo methods in atmospheric optics*, Springer series in optical sciences. (Springer, Berlin Heidelberg New York) No. 12, (1980).
2. RF Cahalan, W Ridgway, WJ Wiscombe, S Gollmer, Harshvardhan, Independent pixel and monte carlo estimates of stratocumulus albedo. *J. Atmospheric Sci.* **51**, 3776 – 3790 (1994).
3. MF Modest, *Radiative Heat Transfer*. (Elsevier), (2013).
4. S Prahl, miepython: A python library for mie scattering calculations (2025).
5. DJ Segelstein, The complex refractive index of water. Ph.d. thesis (University of Missouri–Kansas City) (1981).
